# Supplementary material for: Prevalence and associated risk factors of tinnitus among Palestinian adolescents aged 15–18: A cross-sectional study
Source: PLoS One. 2026 Mar 9;21(3):e0344420. doi: 10.1371/journal.pone.0344420 (PMC12970918; doi:10.1371/journal.pone.0344420)
Supplement: S1 File — (DOCX) [file pone.0344420.s001.docx]

Study questionnaire and spss coding (red)

- Age: Q1
- 15 1
- 16 2
- 17 3
- 18 4
- Gender: Q2
- Male 1
- Female 2
- What is your height? ………………….. Q3
- What is your weight?..................... Q4

BMI = weight (kg) / [height (m)]2

- Underweight >18.5
- Normal weight 18.5 - 24.9
- Overweight 25 - 29.9
- Obesity ≥ 30
- Do you use your phone a lot? Q5
- Yes 1
- No 2
- Not much 3
- Which hand do you use for writing? Q6
- Right 1
- Left 2
- Both 3
- City Q7
- Hebron 1
- Nablus 2
- Jenin 3
- Ramallah 4
- Bethlehem 5
- How many first-degree relatives (parents, children, siblings) do you know have tinnitus? (You may select more than one option.) Q8a
- Mother 1
- Father 2
- Brothers 3
- Sisters 4
- I don’t know 5
- None 6
- If you have relatives with tinnitus, specify the number:……………… Q8b
- ≤ 3
- 4 – 6
- ≥ 7
- Do you experience dizziness? Q9
- Yes, less than once a year 1
- Yes, more than once a year 2
- No 3
- Have you been diagnosed with any ear-related condition? (You may select more than one option.) Q10
- Acoustic trauma due to sudden loud noise 1
- Middle ear infection due to external pressure 2
- Age-related hearing loss 3
- Sudden hearing loss 4
- Acoustic neuroma 5
- Chronic ear infection 6
- Otosclerosis 7
- Middle ear infection or Eustachian tube dysfunction 8
- Eardrum perforation 9
- Hearing loss due to other reasons 10
- None 11
- Other... 12
- Have you undergone any of the following procedures? (You may select more than one option.) Q11
- Ear surgery 1
- Dental procedure (filling removal, dental implants, prolonged dental surgery) 2
- Neurosurgery 3
- Spinal tap (lumbar puncture) 4
- Chemotherapy 5
- Radiation therapy for the head and neck 6
- Electroconvulsive therapy 7
- None 8
- Other... 9 Nasal polypectomy, Tonsillectomy, Pulmonary Laceration, Hand Fracture Sleeve Gastrectomy
- During the past week, have external sounds seemed too loud or uncomfortable for you while sounding normal to others around you? (Note: External sounds refer to any sound other than tinnitus, such as environmental noise, speech, or music.) Q12
- Yes 1
- No 2
- Do you currently experience any difficulty in hearing, such as understanding speech in noisy environments? Q13
- No 1
- Yes, I cannot hear at all 2
- Slight difficulty 3
- Do you use any of the following devices? (You may select more than one option.) Q14
- Hearing aid 1
- Cochlear implant 2
- Sound generator 3
- Combined device (hearing aid and sound generator in one device) 4
- None 5
- Do you experience any of the following symptoms? (You may select more than one option.) Q15
- Headache 1
- Neck pain 2
- Ear pain 3
- Jaw joint pain 4
- Facial pain 5
- None 6
- Other . . . Shoulder pain 7
- Do you suffer from any of the following conditions? (You may select more than one option.)
- Headache
- Neck pain
- Ear pain
- Jaw joint pain
- Facial pain
- None
- Have you been diagnosed with any of the following conditions by a doctor? (You may select more than one option. If none apply, you may skip this section.) Q16
- Oral conditions: Q16a
- Jaw joint pain 1
- Dental problems 2
- Neurological conditions: Q16b
- Meningitis 1
- Multiple sclerosis 2
- Epilepsy 3
- Stroke 4
- Other cerebrovascular diseases 5
- Psychological conditions: Q16c
- Anxiety, excessive stress 1
- Depression 2
- Emotional trauma 3
- Sleep disorders: Q16d
- Difficulty falling asleep 1
- Difficulty staying asleep 2
- Cardiovascular conditions: Q16e
- Low blood pressure 1
- High blood pressure 2
- Myocardial infarction (heart attack) 3
- Endocrine and metabolic conditions: Q16f
- Thyroid dysfunction 1
- Diabetes 2
- High cholesterol 3
- Rheumatic and autoimmune disorders: Q16g
- Rheumatoid arthritis 1
- Lupus (facial rash) 2
- Ear, nose, and throat conditions: Q16h
- Chronic sinusitis 1
- Deviated nasal septum 2
- Other conditions: Q17i
- Anemia 1
- Balance disorder 2
- Gastroesophageal reflux disease (GERD) 3
- If you have any other conditions, please specify: ………….. Q16j

Adenoid, Asthma, Epistaxis, Familial Mediterranean Fever, HSV, Helicobacter pylori, Iron Deficiency, Leukemia, Migraine, OCD, Schizophrenia, parkinson's disease

- Tinnitus refers to the perception of noise inside your head or ear (such as ringing) without an external sound source. Over the past year, have you experienced tinnitus in one or both ears that lasted more than 5 minutes each time? Q17
- Yes, most or all of the time 1
- Yes, frequently 2
- Yes, occasionally 3
- No, not in the past year 4
- Never 5
- I don’t know 6

Tinnitus characteristics:

Thank you for completing the previous section. For the following questions, please provide the answers that best describe your tinnitus and its relationship to other conditions. You may select more than one option for some questions.

- On average, how often do you experience tinnitus? Q18
- Daily or almost daily 1
- About weekly 2
- About monthly 3
- Every few months 4
- Yearly 5
- Which best describes your tinnitus throughout the day? Q19
- Constant: You can always or usually hear it in a quiet room 1
- Intermittent: It comes and goes; you cannot always hear it in a quiet room 2
- When did your tinnitus start? ……….. Q20
- 1–12 months 1
- 13–24 month 2
- 25–36 month 3
- > 36 months 4
- I don’t know 5
- If you have mentioned conditions or procedures in the previous section, please specify whether they occurred before, after, or around the same time your tinnitus started? …….. Q21
- Before 1
- After 2
- Around the same time 3
- I don’t know 4
- Was the onset of your tinnitus related to any of the following? (You may select more than one option.) Q22
- Exposure to loud sounds 1
- Change in hearing 2
- Exposure to changes in surrounding pressure (such as flying or driving) 3
- Flu, common cold, or other infections 4
- Feeling of fullness or pressure in the ears 5
- Anxiety 6
- Head injury 7
- Neck injury 8
- None 9
- Other... 10
- Were you taking any of the following medications around the time your tinnitus began? (You may select more than one option.) Q23
- Aspirin 1
- Pain relievers 2
- Antibiotics 3
- Quinine (used for muscle cramps, malaria, anti-inflammatory purposes) 4
- Diuretics 5
- Antidepressants 6
- None 7
- I don’t know 8
- Other... 9
- What does your tinnitus sound like? Q24
- Tonal (continuous sound with varying frequencies) 1
- Noise-like 2
- Music-like 3
- Sounds like a cricket 4
- Buzzing 5
- Other... 6 None, I don't know
- Where do you feel your tinnitus? Q25
- Right ear 1
- Left ear 2
- Both ears, worse in the right 3
- Both ears, worse in the left 4
- Both ears equally 5
- Inside the head 6
- I don’t know 7
- Other... 8, None
- Is your tinnitus rhythmic? Q26
- Yes, it follows my heartbeat (it may be checked by feeling the pulse at the same time as the tinnitus) 1
- Yes, it follows my breathing 2
- Yes, it follows head, neck, jaw, or facial muscle movements 4
- Other... 5 No, NO, None, I don't know
- Has your doctor ever heard your tinnitus? Q27
- Yes 1
- No 2
- Within a year of developing tinnitus, did you visit a family doctor or a healthcare specialist at a clinic or hospital for your tinnitus? Q28
- Yes, 5 or more visits 1
- Yes, 2 to 4 visits 2
- Yes, only one visit 3
- No, never 4
- I don’t know 5
- Are you currently receiving any of the following treatments for your tinnitus? (You may select more than one option.) Q29
- Psychological therapy 1
- Audiological therapy 2
- Physical therapy 3
- Self-management (dietary supplements, support groups, relaxation) 4
- None 5
- Other... 6
