# Supplementary material for: Prevalence and associated risk factors of tinnitus among Palestinian adolescents aged 15–18: A cross-sectional study
Source: PLoS One. 2026 Mar 9;21(3):e0344420. doi: 10.1371/journal.pone.0344420 (PMC12970918; doi:10.1371/journal.pone.0344420)
Supplement: S2 File — (PDF) [file pone.0344420.s002.pdf]

Paragraph

Frequencies

Notes

Output Created		09-APR-2025 01:49:18
Comments		
Input	Data	C:\Users\Dell\OneDrive - Universiti Kebangsaan Malaysia\Tinnitus\Tinnitus Spss.sav
	Active Dataset	DataSet1
	Filter	<none>
	Weight	<none>
	Split File	<none>
	N of Rows in Working Data File	1131
Missing Value Handling	Definition of Missing	User-defined missing values are treated as missing.
	Cases Used	Statistics are based on all cases with valid data.
Syntax		FREQUENCIES VARIABLES=TinnitusGroup /PERCENTILES=100.0 /ORDER=ANALYSIS.
Resources	Processor Time	00:00:00.03
	Elapsed Time	00:00:00.01

[DataSet1] C:\Users\Dell\OneDrive - Universiti Kebangsaan Malaysia\Tinnitus\Tinnitus Spss.sav

Statistics

TinnitusGroup

N	Valid	1131
	Missing	0
Percentiles	100	1.0000

TinnitusGroup

		Frequency	Percent	Valid Percent	Cumulative Percent
Valid	.00	599	53.0	53.0	53.0
	1.00	532	47.0	47.0	100.0
	Total	1131	100.0	100.0	

Paragraph

Frequencies

Notes

Output Created		09-APR-2025 01:09:26
Comments		
Input	Data	C:\Users\Dell\OneDrive - Universiti Kebangsaan Malaysia\Tinnitus\Tinnitus Spss.sav
	Active Dataset	DataSet1
	Filter	<none>
	Weight	<none>
	Split File	<none>
	N of Rows in Working Data File	1131
Missing Value Handling	Definition of Missing	User-defined missing values are treated as missing.
	Cases Used	Statistics are based on all cases with valid data.

Notes

Syntax	FREQUENCIES VARIABLES=Q9 MiddleEarDueToQ10 AgeRelatedHLQ10 AcousticTraumaQ10 SuddenHLQ10 AcousticNeuromaQ10 ChronicEarInfectionQ10 OtosclerosisQ10 MiddleEarOrEustachianQ1 0 EardrumPerforationQ10 HLDueToOtherReasonsQ1 0 NNoneQ10 EarSurgeryQ11 DentalProcedureQ11 NeurosurgeryQ11 SpinalTapQ11 ChemotherapyQ11 RadiationTherapyQ11 ElectroconvulsiveTherapyQ 11 NNoneQ11 NasalpolypectomyQ11 TonsillectomyQ11 PulmonaryLacerationQ11 HandFractureQ11 SleeveGastrectomyQ11 Q12 Q13 Q14 HearingAidQ14 CochlearImplantQ14 SoundGeneratorQ14 CombinedDeviceQ14 NoneQ14 HeadacheQ15 NeckPainQ15 EarPainQ15 JawJointPainQ15 FacialPainQ15 NNoneQ15 ShoulderPainQ15 JawJointPainQ16a DentalProblomsQ16a MeningitisQ16b MultipleSclerosisQ16b EpilepsyQ16b StrokeQ16b OtherCerebrovascularDise asesQ16b AnxietyQ16c DepressionQ16c EmotionalTraumaQ16c DifficultyFallingQ16d DifficultyStayingQ16d LowBPQ16e HighBPQ16e MyocardialInfarctionQ16e ThyroidDysfunctionQ16f DiabetesQ16f HighCholesterolQ16f LupusQ16g RheumatoidArthritisQ16g ChronicSinusitisQ16h<br><br>DeviatedNasalSeptumQ16 h AnemiaQ16i BalanceDisorderQ16i GERDGroup AdenoidQ16j AsthmaQ16j EpistaxisQ16j FamilialMediterraneanFeve rQ16j HSVQ16j HelicobacterPyloriQ16j IronDeficiencyQ16j LeukemiaQ16j

Notes

Resources	Processor Time	00:00:00.09
	Elapsed Time	00:00:00.09

Statistics

		Do you experience dizziness? Q9	MiddleEarDueToQ10	AgeRelatedHLQ10	AcousticTraumaQ10
N	Valid	1131	1131	1131	1131
	Missing	0	0	0	0
Percentiles	100		1.0000	1.0000	1.0000

Statistics

		SuddenHLQ10	AcousticNeuromaQ10	ChronicEarInfectionQ10	OtosclerosisQ10
N	Valid	1131	1131	1131	1131
	Missing	0	0	0	0
Percentiles	100	1.0000	1.0000	1.0000	1.0000

Statistics

		MiddleEarOrEustachianQ10	EardrumPerforationQ10	HLDueToOtherReasonsQ10	NNoneQ10
N	Valid	1131	1131	1131	1131
	Missing	0	0	0	0
Percentiles	100	1.0000	1.0000	1.0000	1.0000

Statistics

		EarSurgeryQ11	DentalProcedureQ11	NeurosurgeryQ11	SpinalTapQ11
N	Valid	1131	1131	1131	1131
	Missing	0	0	0	0
Percentiles	100	1.0000	1.0000	1.0000	1.0000

Statistics

		ChemotherapyQ11	RadiationTherapyQ11	ElectroconvulsiveTherapyQ11	NNoneQ11
N	Valid	1131	1131	1131	1131
	Missing	0	0	0	0
Percentiles	100	1.0000	1.0000	1.0000	1.0000

Statistics

		NasalpolypectomyQ11	TonsillectomyQ11	PulmonaryLacerationQ11	HandFractureQ11
N	Valid	1131	1131	1131	1131
	Missing	0	0	0	0
Percentiles	100	1.0000	1.0000	1.0000	1.0000

Statistics

		SleeveGastrectomyQ11	During the past week, have external sounds seemed too loud or uncomfortable for you while sounding normal to others around you?Q12	Do you currently experience any difficulty in hearing, such as understanding speech in noisy environments?Q13	Do you use any of the following devices?Q14
N	Valid	1131	1131	1131	1131
	Missing	0	0	0	0
Percentiles	100	1.0000			

Statistics

		HearingAidQ14	CochlearImplantQ14	SoundGeneratorQ14	CombinedDeviceQ14	NoneQ14
N	Valid	1131	1131	1131	1131	1131
	Missing	0	0	0	0	0
Percentiles	100	1.0000	1.0000	1.0000	1.0000	1.0000

Statistics

		HeadacheQ15	NeckPainQ15	EarPainQ15	JawJointPainQ15	FacialPainQ15
N	Valid	1131	1131	1131	1131	1131
	Missing	0	0	0	0	0
Percentiles	100	1.0000	1.0000	1.0000	1.0000	1.0000

Statistics

		NNoneQ15	ShoulderPainQ15	JawJointPainQ16a	DentalProbloms Q16a
N	Valid	1131	1131	1131	1131
	Missing	0	0	0	0
Percentiles	100	1.0000	1.0000	1.0000	1.0000

Statistics

		MeningitisQ16b	MultipleSclerosisQ16b	EpilepsyQ16b	StrokeQ16b
N	Valid	1131	1131	1131	1131
	Missing	0	0	0	0
Percentiles	100	1.0000	1.0000	1.0000	1.0000

Statistics

		OtherCerebrovascularDiseasesQ16b	AnxietyQ16c	DepressionQ16c	EmotionalTraumaQ16c
N	Valid	1131	1131	1131	1131
	Missing	0	0	0	0
Percentiles	100	1.0000	1.0000	1.0000	1.0000

Statistics

		DifficultyFallingAsleepQ16d	DifficultyStayingAsleepQ16d	LowBPQ16e	HighBPQ16e	MyocardialInfarctionQ16e
N	Valid	1131	1131	1131	1131	1131
	Missing	0	0	0	0	0
Percentiles	100	1.0000	1.0000	1.0000	1.0000	1.0000

Statistics

		ThyroidDysfunctionQ16f	DiabetesQ16f	HighCholesterolQ16f	LupusQ16g	RheumatoidArthritisQ16g
N	Valid	1131	1131	1131	1131	1131
	Missing	0	0	0	0	0
Percentiles	100	1.0000	1.0000	1.0000	1.0000	1.0000

Statistics

		ChronicSinusitisQ16h	DeviatedNasalSeptumQ16h	AnemiaQ16i	BalanceDisorderQ16i	GERDGroup
N	Valid	1131	1131	1131	1131	1131
	Missing	0	0	0	0	0
Percentiles	100	1.0000	1.0000	1.0000	1.0000	1.0000

Statistics

		AdenoidQ16j	AsthmaQ16j	EpistaxisQ16j	FamilialMediterraneanFeverQ16j	HSVQ16j
N	Valid	1131	1131	1131	1131	1131
	Missing	0	0	0	0	0
Percentiles	100	1.0000	1.0000	1.0000	1.0000	1.0000

Statistics

		HelicobacterPyloriQ16j	IronDeficiencyQ16j	LeukemiaQ16j	MigraineQ16j	OCDQ16j
N	Valid	1131	1131	1131	1131	1131
	Missing	0	0	0	0	0
Percentiles	100	1.0000	1.0000	1.0000	1.0000	1.0000

Statistics

		SchizophreniaQ16j	ParkinsonQ16j
N	Valid	1131	1131
	Missing	0	0
Percentiles	100	1.0000	1.0000

Frequency Table

Do you experience dizziness? Q9

		Frequency	Percent	Valid Percent	Cumulative Percent
Valid	No	353	31.2	31.2	31.2
	Yes, less than once a year	295	26.1	26.1	57.3
	Yes, more than once a year	439	38.8	38.8	96.1
	Yes, more then once a year	44	3.9	3.9	100.0
	Total	1131	100.0	100.0	

MiddleEarDueToQ10

		Frequency	Percent	Valid Percent	Cumulative Percent
Valid	.00	1036	91.6	91.6	91.6
	1.00	95	8.4	8.4	100.0
	Total	1131	100.0	100.0	

AgeRelatedHLQ10

		Frequency	Percent	Valid Percent	Cumulative Percent
Valid	.00	1130	99.9	99.9	99.9
	1.00	1	.1	.1	100.0
	Total	1131	100.0	100.0	

AcousticTraumaQ10

		Frequency	Percent	Valid Percent	Cumulative Percent
Valid	.00	1090	96.4	96.4	96.4
	1.00	41	3.6	3.6	100.0
	Total	1131	100.0	100.0	

SuddenHLQ10

		Frequency	Percent	Valid Percent	Cumulative Percent
Valid	.00	1124	99.4	99.4	99.4
	1.00	7	.6	.6	100.0
	Total	1131	100.0	100.0	

AcousticNeuromaQ10

		Frequency	Percent	Valid Percent	Cumulative Percent
Valid	.00	1130	99.9	99.9	99.9
	1.00	1	.1	.1	100.0
	Total	1131	100.0	100.0	

ChronicEarInfectionQ10

		Frequency	Percent	Valid Percent	Cumulative Percent
Valid	.00	1104	97.6	97.6	97.6
	1.00	27	2.4	2.4	100.0
	Total	1131	100.0	100.0	

OtosclerosisQ10

		Frequency	Percent	Valid Percent	Cumulative Percent
Valid	.00	1130	99.9	99.9	99.9
	1.00	1	.1	.1	100.0
	Total	1131	100.0	100.0	

MiddleEarOrEustachianQ10

		Frequency	Percent	Valid Percent	Cumulative Percent
Valid	.00	1106	97.8	97.8	97.8
	1.00	25	2.2	2.2	100.0
	Total	1131	100.0	100.0	

EardrumPerforationQ10

		Frequency	Percent	Valid Percent	Cumulative Percent
Valid	.00	1111	98.2	98.2	98.2
	1.00	20	1.8	1.8	100.0
	Total	1131	100.0	100.0	

HLDueToOtherReasonsQ10

		Frequency	Percent	Valid Percent	Cumulative Percent
Valid	.00	1114	98.5	98.5	98.5
	1.00	17	1.5	1.5	100.0
	Total	1131	100.0	100.0	

NNoneQ10

		Frequency	Percent	Valid Percent	Cumulative Percent
Valid	.00	197	17.4	17.4	17.4
	1.00	934	82.6	82.6	100.0
	Total	1131	100.0	100.0	

EarSurgeryQ11

		Frequency	Percent	Valid Percent	Cumulative Percent
Valid	.00	1105	97.7	97.7	97.7
	1.00	26	2.3	2.3	100.0
	Total	1131	100.0	100.0	

DentalProcedureQ11

		Frequency	Percent	Valid Percent	Cumulative Percent
Valid	.00	903	79.8	79.8	79.8
	1.00	228	20.2	20.2	100.0
	Total	1131	100.0	100.0	

NeurosurgeryQ11

		Frequency	Percent	Valid Percent	Cumulative Percent
Valid	.00	1118	98.9	98.9	98.9
	1.00	13	1.1	1.1	100.0
	Total	1131	100.0	100.0	

SpinalTapQ11

		Frequency	Percent	Valid Percent	Cumulative Percent
Valid	.00	1121	99.1	99.1	99.1
	1.00	10	.9	.9	100.0
	Total	1131	100.0	100.0	

ChemotherapyQ11

		Frequency	Percent	Valid Percent	Cumulative Percent
Valid	.00	1126	99.6	99.6	99.6
	1.00	5	.4	.4	100.0
	Total	1131	100.0	100.0	

RadiationTherapyQ11

		Frequency	Percent	Valid Percent	Cumulative Percent
Valid	.00	1120	99.0	99.0	99.0
	1.00	11	1.0	1.0	100.0
	Total	1131	100.0	100.0	

ElectroconvulsiveTherapyQ11

		Frequency	Percent	Valid Percent	Cumulative Percent
Valid	.00	1126	99.6	99.6	99.6
	1.00	5	.4	.4	100.0
	Total	1131	100.0	100.0	

NNoneQ11

		Frequency	Percent	Valid Percent	Cumulative Percent
Valid	.00	285	25.2	25.2	25.2
	1.00	846	74.8	74.8	100.0
	Total	1131	100.0	100.0	

NasalpolypectomyQ11

		Frequency	Percent	Valid Percent	Cumulative Percent
Valid	.00	1127	99.6	99.6	99.6
	1.00	4	.4	.4	100.0
	Total	1131	100.0	100.0	

TonsillectomyQ11

		Frequency	Percent	Valid Percent	Cumulative Percent
Valid	.00	1114	98.5	98.5	98.5
	1.00	17	1.5	1.5	100.0
	Total	1131	100.0	100.0	

PulmonaryLacerationQ11

		Frequency	Percent	Valid Percent	Cumulative Percent
Valid	.00	1130	99.9	99.9	99.9
	1.00	1	.1	.1	100.0
	Total	1131	100.0	100.0	

HandFractureQ11

		Frequency	Percent	Valid Percent	Cumulative Percent
Valid	.00	1130	99.9	99.9	99.9
	1.00	1	.1	.1	100.0
	Total	1131	100.0	100.0	

SleeveGastrectomyQ11

		Frequency	Percent	Valid Percent	Cumulative Percent
Valid	.00	1130	99.9	99.9	99.9
	1.00	1	.1	.1	100.0
	Total	1131	100.0	100.0	

During the past week, have external sounds seemed too loud or uncomfortable for you while sounding normal to others around you?Q12

		Frequency	Percent	Valid Percent	Cumulative Percent
Valid	No	739	65.3	65.3	65.3
	Yes	392	34.7	34.7	100.0
	Total	1131	100.0	100.0	

Do you currently experience any difficulty in hearing, such as understanding speech in noisy environments?Q13

		Frequency	Percent	Valid Percent	Cumulative Percent
Valid	No	619	54.7	54.7	54.7
	Slight difficulty	479	42.4	42.4	97.1
	Yes,I con't hear at all	33	2.9	2.9	100.0
	Total	1131	100.0	100.0	

Do you use any of the following devices?Q14

		Frequency	Percent	Valid Percent	Cumulative Percent
Valid	Cochlear implant	1	.1	.1	.1
	Combined device (hearing aid and sound generator in one device)	3	.3	.3	.4
	Hearing aid	8	.7	.7	1.1
	None	1117	98.8	98.8	99.8
	Sound generator	2	.2	.2	100.0
	Total	1131	100.0	100.0	

HearingAidQ14

		Frequency	Percent	Valid Percent	Cumulative Percent
Valid	.00	1123	99.3	99.3	99.3
	1.00	8	.7	.7	100.0
	Total	1131	100.0	100.0	

CochlearImplantQ14

		Frequency	Percent	Valid Percent	Cumulative Percent
Valid	.00	1130	99.9	99.9	99.9
	1.00	1	.1	.1	100.0
	Total	1131	100.0	100.0	

SoundGeneratorQ14

		Frequency	Percent	Valid Percent	Cumulative Percent
Valid	.00	1129	99.8	99.8	99.8
	1.00	2	.2	.2	100.0
	Total	1131	100.0	100.0	

CombinedDeviceQ14

		Frequency	Percent	Valid Percent	Cumulative Percent
Valid	.00	1128	99.7	99.7	99.7
	1.00	3	.3	.3	100.0
	Total	1131	100.0	100.0	

NoneQ14

		Frequency	Percent	Valid Percent	Cumulative Percent
Valid	.00	14	1.2	1.2	1.2
	1.00	1117	98.8	98.8	100.0
	Total	1131	100.0	100.0	

HeadacheQ15

		Frequency	Percent	Valid Percent	Cumulative Percent
Valid	.00	657	58.1	58.1	58.1
	1.00	474	41.9	41.9	100.0
	Total	1131	100.0	100.0	

NeckPainQ15

		Frequency	Percent	Valid Percent	Cumulative Percent
Valid	.00	923	81.6	81.6	81.6
	1.00	208	18.4	18.4	100.0
	Total	1131	100.0	100.0	

EarPainQ15

		Frequency	Percent	Valid Percent	Cumulative Percent
Valid	.00	1019	90.1	90.1	90.1
	1.00	112	9.9	9.9	100.0
	Total	1131	100.0	100.0	

JawJointPainQ15

		Frequency	Percent	Valid Percent	Cumulative Percent
Valid	.00	1030	91.1	91.1	91.1
	1.00	101	8.9	8.9	100.0
	Total	1131	100.0	100.0	

FacialPainQ15

		Frequency	Percent	Valid Percent	Cumulative Percent
Valid	.00	1090	96.4	96.4	96.4
	1.00	41	3.6	3.6	100.0
	Total	1131	100.0	100.0	

NNoneQ15

		Frequency	Percent	Valid Percent	Cumulative Percent
Valid	.00	558	49.3	49.3	49.3
	1.00	573	50.7	50.7	100.0
	Total	1131	100.0	100.0	

ShoulderPainQ15

		Frequency	Percent	Valid Percent	Cumulative Percent
Valid	.00	1126	99.6	99.6	99.6
	1.00	5	.4	.4	100.0
	Total	1131	100.0	100.0	

JawJointPainQ16a

		Frequency	Percent	Valid Percent	Cumulative Percent
Valid	.00	1025	90.6	90.6	90.6
	1.00	106	9.4	9.4	100.0
	Total	1131	100.0	100.0	

DentalProblomsQ16a

		Frequency	Percent	Valid Percent	Cumulative Percent
Valid	.00	774	68.4	68.4	68.4
	1.00	357	31.6	31.6	100.0
	Total	1131	100.0	100.0	

MeningitisQ16b

		Frequency	Percent	Valid Percent	Cumulative Percent
Valid	.00	1120	99.0	99.0	99.0
	1.00	11	1.0	1.0	100.0
	Total	1131	100.0	100.0	

MultipleSclerosisQ16b

		Frequency	Percent	Valid Percent	Cumulative Percent
Valid	.00	1123	99.3	99.3	99.3
	1.00	8	.7	.7	100.0
	Total	1131	100.0	100.0	

EpilepsyQ16b

		Frequency	Percent	Valid Percent	Cumulative Percent
Valid	.00	1107	97.9	97.9	97.9
	1.00	24	2.1	2.1	100.0
	Total	1131	100.0	100.0	

StrokeQ16b

		Frequency	Percent	Valid Percent	Cumulative Percent
Valid	.00	1128	99.7	99.7	99.7
	1.00	3	.3	.3	100.0
	Total	1131	100.0	100.0	

OtherCerebrovascularDiseasesQ16b

		Frequency	Percent	Valid Percent	Cumulative Percent
Valid	.00	1122	99.2	99.2	99.2
	1.00	9	.8	.8	100.0
	Total	1131	100.0	100.0	

AnxietyQ16c

		Frequency	Percent	Valid Percent	Cumulative Percent
Valid	.00	705	62.3	62.3	62.3
	1.00	426	37.7	37.7	100.0
	Total	1131	100.0	100.0	

DepressionQ16c

		Frequency	Percent	Valid Percent	Cumulative Percent
Valid	.00	990	87.5	87.5	87.5
	1.00	141	12.5	12.5	100.0
	Total	1131	100.0	100.0	

EmotionalTraumaQ16c

		Frequency	Percent	Valid Percent	Cumulative Percent
Valid	.00	1049	92.7	92.7	92.7
	1.00	82	7.3	7.3	100.0
	Total	1131	100.0	100.0	

DifficultyFallingAsleepQ16d

		Frequency	Percent	Valid Percent	Cumulative Percent
Valid	.00	759	67.1	67.1	67.1
	1.00	372	32.9	32.9	100.0
	Total	1131	100.0	100.0	

DifficultyStayingAsleepQ16d

		Frequency	Percent	Valid Percent	Cumulative Percent
Valid	.00	951	84.1	84.1	84.1
	1.00	180	15.9	15.9	100.0
	Total	1131	100.0	100.0	

LowBPQ16e

		Frequency	Percent	Valid Percent	Cumulative Percent
Valid	.00	1077	95.2	95.2	95.2
	1.00	54	4.8	4.8	100.0
	Total	1131	100.0	100.0	

HighBPQ16e

		Frequency	Percent	Valid Percent	Cumulative Percent
Valid	.00	1096	96.9	96.9	96.9
	1.00	35	3.1	3.1	100.0
	Total	1131	100.0	100.0	

MyocardialInfarctionQ16e

		Frequency	Percent	Valid Percent	Cumulative Percent
Valid	.00	1129	99.8	99.8	99.8
	1.00	2	.2	.2	100.0
	Total	1131	100.0	100.0	

ThyroidDysfunctionQ16f

		Frequency	Percent	Valid Percent	Cumulative Percent
Valid	.00	1107	97.9	97.9	97.9
	1.00	24	2.1	2.1	100.0
	Total	1131	100.0	100.0	

DiabetesQ16f

		Frequency	Percent	Valid Percent	Cumulative Percent
Valid	.00	1121	99.1	99.1	99.1
	1.00	10	.9	.9	100.0
	Total	1131	100.0	100.0	

HighCholesterolQ16f

		Frequency	Percent	Valid Percent	Cumulative Percent
Valid	.00	1120	99.0	99.0	99.0
	1.00	11	1.0	1.0	100.0
	Total	1131	100.0	100.0	

LupusQ16g

		Frequency	Percent	Valid Percent	Cumulative Percent
Valid	.00	1122	99.2	99.2	99.2
	1.00	9	.8	.8	100.0
	Total	1131	100.0	100.0	

RheumatoidArthritisQ16g

		Frequency	Percent	Valid Percent	Cumulative Percent
Valid	.00	1109	98.1	98.1	98.1
	1.00	22	1.9	1.9	100.0
	Total	1131	100.0	100.0	

ChronicSinusitisQ16h

		Frequency	Percent	Valid Percent	Cumulative Percent
Valid	.00	1014	89.7	89.7	89.7
	1.00	117	10.3	10.3	100.0
	Total	1131	100.0	100.0	

DeviatedNasalSeptumQ16h

		Frequency	Percent	Valid Percent	Cumulative Percent
Valid	.00	1085	95.9	95.9	95.9
	1.00	46	4.1	4.1	100.0
	Total	1131	100.0	100.0	

AnemiaQ16i

		Frequency	Percent	Valid Percent	Cumulative Percent
Valid	.00	1051	92.9	92.9	92.9
	1.00	80	7.1	7.1	100.0
	Total	1131	100.0	100.0	

BalanceDisorderQ16i

		Frequency	Percent	Valid Percent	Cumulative Percent
Valid	.00	1052	93.0	93.0	93.0
	1.00	79	7.0	7.0	100.0
	Total	1131	100.0	100.0	

GERDGroup

		Frequency	Percent	Valid Percent	Cumulative Percent
Valid	.00	1104	97.6	97.6	97.6
	1.00	27	2.4	2.4	100.0
	Total	1131	100.0	100.0	

AdenoidQ16j

		Frequency	Percent	Valid Percent	Cumulative Percent
Valid	.00	1129	99.8	99.8	99.8
	1.00	2	.2	.2	100.0
	Total	1131	100.0	100.0	

AsthmaQ16j

		Frequency	Percent	Valid Percent	Cumulative Percent
Valid	.00	1128	99.7	99.7	99.7
	1.00	3	.3	.3	100.0
	Total	1131	100.0	100.0	

EpistaxisQ16j

		Frequency	Percent	Valid Percent	Cumulative Percent
Valid	.00	1129	99.8	99.8	99.8
	1.00	2	.2	.2	100.0
	Total	1131	100.0	100.0	

FamilialMediterraneanFeverQ16j

		Frequency	Percent	Valid Percent	Cumulative Percent
Valid	.00	1130	99.9	99.9	99.9
	1.00	1	.1	.1	100.0
	Total	1131	100.0	100.0	

HSVQ16j

		Frequency	Percent	Valid Percent	Cumulative Percent
Valid	.00	1130	99.9	99.9	99.9
	1.00	1	.1	.1	100.0
	Total	1131	100.0	100.0	

HelicobacterPyloriQ16j

		Frequency	Percent	Valid Percent	Cumulative Percent
Valid	.00	1130	99.9	99.9	99.9
	1.00	1	.1	.1	100.0
	Total	1131	100.0	100.0	

IronDeficiencyQ16j

		Frequency	Percent	Valid Percent	Cumulative Percent
Valid	.00	1130	99.9	99.9	99.9
	1.00	1	.1	.1	100.0
	Total	1131	100.0	100.0	

LeukemiaQ16j

		Frequency	Percent	Valid Percent	Cumulative Percent
Valid	.00	1130	99.9	99.9	99.9
	1.00	1	.1	.1	100.0
	Total	1131	100.0	100.0	

MigraineQ16j

		Frequency	Percent	Valid Percent	Cumulative Percent
Valid	.00	1126	99.6	99.6	99.6
	1.00	5	.4	.4	100.0
	Total	1131	100.0	100.0	

OCDQ16j

		Frequency	Percent	Valid Percent	Cumulative Percent
Valid	.00	1130	99.9	99.9	99.9
	1.00	1	.1	.1	100.0
	Total	1131	100.0	100.0	

SchizophreniaQ16j

		Frequency	Percent	Valid Percent	Cumulative Percent
Valid	.00	1130	99.9	99.9	99.9
	1.00	1	.1	.1	100.0
	Total	1131	100.0	100.0	

ParkinsonQ16j

		Frequency	Percent	Valid Percent	Cumulative Percent
Valid	.00	1130	99.9	99.9	99.9
	1.00	1	.1	.1	100.0
	Total	1131	100.0	100.0	

Paragraph

Frequencies

Notes

Output Created		09-APR-2025 01:40:01
Comments		
Input	Data	C:\Users\Dell\OneDrive - Universiti Kebangsaan Malaysia\Tinnitus\Tinnitus Spss - Copy - Copy.sav
	Active Dataset	DataSet1
	Filter	<none>
	Weight	<none>
	Split File	<none>
	N of Rows in Working Data File	532
Missing Value Handling	Definition of Missing	User-defined missing values are treated as missing.
	Cases Used	Statistics are based on all cases with valid data.
Syntax		FREQUENCIES VARIABLES=Q17 Q18 Q19 Q24 Q26 Q25 Q27 /PERCENTILES=100.0 /ORDER=ANALYSIS.
Resources	Processor Time	00:00:00.02
	Elapsed Time	00:00:00.02

[DataSet1] C:\Users\Dell\OneDrive - Universiti Kebangsaan Malaysia\Tinnitus\Tinnitus Spss - Copy - Copy.sav

Statistics

		Tinnitus refers to the perception of noise inside your head or ear (such as ringing) without an external sound source. Over the past year, have you experienced tinnitus in one or both ears that lasted more than 5 minutes each time	On average, how often do you experience tinnitus	Which best describes your tinnitus throughout the day	What does your tinnitus sound likeQ24	Is your tinnitus rhythmicQ26
N	Valid	532	532	532	532	532
	Missing	0	0	0	0	0

Statistics

		Where do you feel your tinnitusQ25	Has your doctor ever heard your tinnitusQ27
N	Valid	532	532
	Missing	0	0

Frequency Table

Tinnitus refers to the perception of noise inside your head or ear (such as ringing) without an external sound source. Over the past year, have you experienced tinnitus in one or both ears that lasted more than 5 minutes each time

		Frequency	Percent	Valid Percent	Cumulative Percent
Valid	I don't know	226	42.5	42.5	42.5
	Yes, frequently	66	12.4	12.4	54.9
	Yes, most or all of the time	26	4.9	4.9	59.8
	Yes, occasionally	214	40.2	40.2	100.0
	Total	532	100.0	100.0	

On average, how often do you experience tinnitus

		Frequency	Percent	Valid Percent	Cumulative Percent
Valid	About mly	72	13.5	13.5	13.5
	About weekly	95	17.9	17.9	31.4
	Daily or almost daily	67	12.6	12.6	44.0
	Every few m	150	28.2	28.2	72.2
	Yearly	148	27.8	27.8	100.0
	Total	532	100.0	100.0	

Which best describes your tinnitus throughout the day

		Frequency	Percent	Valid Percent	Cumulative Percent
Valid	Constant: You can always or usually hear it in a quiet room	174	32.7	32.7	32.7
	Intermittent: It comes and goes; you cannot always hear it in a quiet room	358	67.3	67.3	100.0
	Total	532	100.0	100.0	

What does your tinnitus sound likeQ24

		Frequency	Percent	Valid Percent	Cumulative Percent
Valid	Buzzing	20	3.8	3.8	3.8
	I don't know	2	.4	.4	4.1
	Music-like	31	5.8	5.8	10.0
	Noise-like	142	26.7	26.7	36.7
	None	5	.9	.9	37.6
	Sounds like a cricket	185	34.8	34.8	72.4
	Tonal (continuous sound with varying frequencies)	147	27.6	27.6	100.0
	Total	532	100.0	100.0	

Is your tinnitus rhythmicQ26

		Frequency	Percent	Valid Percent	Cumulative Percent
Valid	I don't know	6	1.1	1.1	1.1
	No	390	73.3	73.3	74.4
	NO	1	.2	.2	74.6
	None	3	.6	.6	75.2
	Yes, it follows head, neck, jaw, or facial muscle movements	53	10.0	10.0	85.2
	Yes, it follows my breathing	34	6.4	6.4	91.5
	Yes, it follows my heartbeat (it may be checked by feeling the pulse at the same time as the tinnitus)	45	8.5	8.5	100.0
	Total	532	100.0	100.0	

Where do you feel your tinnitusQ25

		Frequency	Percent	Valid Percent	Cumulative Percent
Valid	Both ears equally	136	25.6	25.6	25.6
	Both ears, worse in the Left	22	4.1	4.1	29.7
	Both ears, worse in the right	60	11.3	11.3	41.0
	I don't know	123	23.1	23.1	64.1
	Inside the head	73	13.7	13.7	77.8
	Left ear	46	8.6	8.6	86.5
	Left ear Inside the head	1	.2	.2	86.7
	None	4	.8	.8	87.4
	Right ear	67	12.6	12.6	100.0
	Total	532	100.0	100.0	

Has your doctor ever heard your tinnitusQ27

		Frequency	Percent	Valid Percent	Cumulative Percent
Valid	No	500	94.0	94.0	94.0
	Yes	32	6.0	6.0	100.0
	Total	532	100.0	100.0	

Paragraph

Frequencies

Notes

Output Created		09-APR-2025 01:45:31
Comments		
Input	Data	C:\Users\Dell\OneDrive - Universiti Kebangsaan Malaysia\Tinnitus\Tinnitus Spss - Copy - Copy.sav
	Active Dataset	DataSet1
	Filter	<none>
	Weight	<none>
	Split File	<none>
	N of Rows in Working Data File	532
Missing Value Handling	Definition of Missing	User-defined missing values are treated as missing.
	Cases Used	Statistics are based on all cases with valid data.
Syntax		FREQUENCIES VARIABLES=TinnitusStart Q20Group Q21n ExposureLoudSoundsQ22 ChangeInHearingQ22 ExposureToChangesQ22 FluQ22 FeelingFullnessQ22 AnxietyQ22 HeadInjuryQ22 NeckInjuryQ22 NNoneQ22 EarInfactionQ22 IdontknowQ22 AspirinQ23 PainRelieversQ23 AntibioticsQ23 QuinineQ23 DiureticsQ23 AntidepressantsQ23 NNoneQ23 IdontknowQ23 /PERCENTILES=100.0 /ORDER=ANALYSIS.
Resources	Processor Time	00:00:00.02
	Elapsed Time	00:00:00.01

Statistics

		TinnitusStartQ20Group	If you have mentioned conditions or procedures in the previous section, please specify whether they occurred before, after, or around the same time your tinnitus started. Q21	ExposureLoudSoundsQ22	ChangeInHearingQ22
N	Valid	522	60	532	532
	Missing	10	472	0	0
Percentiles	100	5.0000	4.0000	1.0000	1.0000

Statistics

		ExposureToChangesQ22	FluQ22	FeelingFullnessQ22	AnxietyQ22	HeadInjuryQ22
N	Valid	532	532	532	532	532
	Missing	0	0	0	0	0
Percentiles	100	1.0000	1.0000	1.0000	1.0000	1.0000

Statistics

		NeckInjuryQ22	NNoneQ22	EarInfactionQ22	IdontknowQ22	AspirinQ23
N	Valid	532	532	532	532	532
	Missing	0	0	0	0	0
Percentiles	100	1.0000	1.0000	1.0000	1.0000	1.0000

Statistics

		PainRelieversQ 23	AntibioticsQ23	QuinineQ23	DiureticsQ23	Antidepressants Q23
N	Valid	532	532	532	532	532
	Missing	0	0	0	0	0
Percentiles	100	1.0000	1.0000	1.0000	1.0000	1.0000

Statistics

		NNoneQ23	IdontknowQ23
N	Valid	532	532
	Missing	0	0
Percentiles	100	1.0000	1.0000

Frequency Table

TinnitusStartQ20Group

		Frequency	Percent	Valid Percent	Cumulative Percent
Valid	1.00	98	18.4	18.8	18.8
	2.00	72	13.5	13.8	32.6
	3.00	31	5.8	5.9	38.5
	4.00	14	2.6	2.7	41.2
	5.00	307	57.7	58.8	100.0
	Total	522	98.1	100.0	
Missing	System	10	1.9		
Total		532	100.0		

If you have mentioned conditions or procedures in the previous section, please specify whether they occurred before, after, or around the same time your tinnitus started.Q21

		Frequency	Percent	Valid Percent	Cumulative Percent
Valid	1.00	14	2.6	23.3	23.3
	2.00	23	4.3	38.3	61.7
	3.00	6	1.1	10.0	71.7
	4.00	17	3.2	28.3	100.0
	Total	60	11.3	100.0	
Missing	System	472	88.7		
Total		532	100.0		

ExposureLoudSoundsQ22

		Frequency	Percent	Valid Percent	Cumulative Percent
Valid	.00	382	71.8	71.8	71.8
	1.00	150	28.2	28.2	100.0
	Total	532	100.0	100.0	

ChangeInHearingQ22

		Frequency	Percent	Valid Percent	Cumulative Percent
Valid	.00	505	94.9	94.9	94.9
	1.00	27	5.1	5.1	100.0
	Total	532	100.0	100.0	

ExposureToChangesQ22

		Frequency	Percent	Valid Percent	Cumulative Percent
Valid	.00	478	89.8	89.8	89.8
	1.00	54	10.2	10.2	100.0
	Total	532	100.0	100.0	

FluQ22

		Frequency	Percent	Valid Percent	Cumulative Percent
Valid	.00	417	78.4	78.4	78.4
	1.00	115	21.6	21.6	100.0
	Total	532	100.0	100.0	

FeelingFullnessQ22

		Frequency	Percent	Valid Percent	Cumulative Percent
Valid	.00	479	90.0	90.0	90.0
	1.00	53	10.0	10.0	100.0
	Total	532	100.0	100.0	

AnxietyQ22

		Frequency	Percent	Valid Percent	Cumulative Percent
Valid	.00	423	79.5	79.5	79.5
	1.00	109	20.5	20.5	100.0
	Total	532	100.0	100.0	

HeadInjuryQ22

		Frequency	Percent	Valid Percent	Cumulative Percent
Valid	.00	504	94.7	94.7	94.7
	1.00	28	5.3	5.3	100.0
	Total	532	100.0	100.0	

NeckInjuryQ22

		Frequency	Percent	Valid Percent	Cumulative Percent
Valid	.00	523	98.3	98.3	98.3
	1.00	9	1.7	1.7	100.0
	Total	532	100.0	100.0	

NNoneQ22

		Frequency	Percent	Valid Percent	Cumulative Percent
Valid	.00	332	62.4	62.4	62.4
	1.00	200	37.6	37.6	100.0
	Total	532	100.0	100.0	

EarInfactionQ22

		Frequency	Percent	Valid Percent	Cumulative Percent
Valid	.00	530	99.6	99.6	99.6
	1.00	2	.4	.4	100.0
	Total	532	100.0	100.0	

IdontknowQ22

		Frequency	Percent	Valid Percent	Cumulative Percent
Valid	.00	528	99.2	99.2	99.2
	1.00	4	.8	.8	100.0
	Total	532	100.0	100.0	

AspirinQ23

		Frequency	Percent	Valid Percent	Cumulative Percent
Valid	.00	522	98.1	98.1	98.1
	1.00	10	1.9	1.9	100.0
	Total	532	100.0	100.0	

PainRelieversQ23

		Frequency	Percent	Valid Percent	Cumulative Percent
Valid	.00	443	83.3	83.3	83.3
	1.00	89	16.7	16.7	100.0
	Total	532	100.0	100.0	

AntibioticsQ23

		Frequency	Percent	Valid Percent	Cumulative Percent
Valid	.00	483	90.8	90.8	90.8
	1.00	49	9.2	9.2	100.0
	Total	532	100.0	100.0	

QuinineQ23

		Frequency	Percent	Valid Percent	Cumulative Percent
Valid	.00	522	98.1	98.1	98.1
	1.00	10	1.9	1.9	100.0
	Total	532	100.0	100.0	

DiureticsQ23

		Frequency	Percent	Valid Percent	Cumulative Percent
Valid	.00	529	99.4	99.4	99.4
	1.00	3	.6	.6	100.0
	Total	532	100.0	100.0	

AntidepressantsQ23

		Frequency	Percent	Valid Percent	Cumulative Percent
Valid	.00	525	98.7	98.7	98.7
	1.00	7	1.3	1.3	100.0
	Total	532	100.0	100.0	

NNoneQ23

		Frequency	Percent	Valid Percent	Cumulative Percent
Valid	.00	217	40.8	40.8	40.8
	1.00	315	59.2	59.2	100.0
	Total	532	100.0	100.0	

IdontknowQ23

		Frequency	Percent	Valid Percent	Cumulative Percent
Valid	.00	444	83.5	83.5	83.5
	1.00	88	16.5	16.5	100.0
	Total	532	100.0	100.0	

Paragraph

Frequencies

Notes

Output Created		09-APR-2025 01:47:08
Comments		
Input	Data	C:\Users\Dell\OneDrive - Universiti Kebangsaan Malaysia\Tinnitus\Tinnitus Spss - Copy - Copy.sav
	Active Dataset	DataSet1
	Filter	<none>
	Weight	<none>
	Split File	<none>
	N of Rows in Working Data File	532
Missing Value Handling	Definition of Missing	User-defined missing values are treated as missing.
	Cases Used	Statistics are based on all cases with valid data.
Syntax		FREQUENCIES VARIABLES=Q28 Q29 /PERCENTILES=100.0 /ORDER=ANALYSIS.
Resources	Processor Time	00:00:00.02
	Elapsed Time	00:00:00.03

Statistics

		Within a year of developing tinnitus, did you visit a family doctor or a healthcare specialist at a clinic or hospital for your tinnitusQ28	Are you currently receiving any of the following treatments for your tinnitusQ29
N	Valid	532	532
	Missing	0	0

Frequency Table

Within a year of developing tinnitus, did you visit a family doctor or a healthcare specialist at a clinic or hospital for your tinnitusQ28

		Frequency	Percent	Valid Percent	Cumulative Percent
Valid	I don't know	93	17.5	17.5	17.5
	No, never	379	71.2	71.2	88.7
	Yes, 2 to 4 visits	18	3.4	3.4	92.1
	Yes, 5 or more visits	8	1.5	1.5	93.6
	Yes, only one visit	34	6.4	6.4	100.0
	Total	532	100.0	100.0	

Are you currently receiving any of the following treatments for your tinnitusQ29

		Frequency	Percent	Valid Percent	Cumulative Percent
Valid	Audiological therapy	5	.9	.9	.9
	None	504	94.7	94.7	95.7
	Physical therapy	2	.4	.4	96.1
	Psychological therapy	5	.9	.9	97.0
	Self-management (dietary supplements, support groups, relaxation)	16	3.0	3.0	100.0
	Total	532	100.0	100.0	
