# Supplementary material for: Prevalence and associated risk factors of tinnitus among Palestinian adolescents aged 15–18: A cross-sectional study
Source: PLoS One. 2026 Mar 9;21(3):e0344420. doi: 10.1371/journal.pone.0344420 (PMC12970918; doi:10.1371/journal.pone.0344420)
Supplement: Attachment — Submitted filename: 12-01-2026 Responses.docx [file pone.0344420.s004.docx]

Dear editor,

Thank you for your time and we are grateful to the respected reviewers for their valuable comments which we believe they enhanced the manuscript. Kindly find below our responses. We highlighted the responses in the revised manuscript in red color.

Kind regards,

Malil Alqub, corresponding author, on behalf of all authors

Reviewer #1: Thank you very much for the opportunity to review this article. I find it highly interesting, and its findings undoubtedly warrant attention from public health policymakers.

We sincerely thank the reviewer for this positive assessment of the study’s relevance.

The assumption of a 50% prevalence for the sample size calculation (which is striking because the prevalence in adults is 30%) is well justified because there were no previous regional data for the specific target population. It is understood that they assumed that tinnitus may have more pronounced risk factors in young people (e.g., exposure to loud music, academic stress levels, etc.). Without specific data for this age group, the researchers took the most rigorous and conservative route from a statistical point of view.

We thank the reviewer for acknowledging and clearly articulating the rationale behind our sample size calculation. As noted, in the absence of prior regional data for Palestinian adolescents, adopting a 50% prevalence estimate was intended to ensure a conservative and statistically rigorous approach.

The only significant methodological weakness of this study is the use of convenience sampling, which introduces a possible selection bias and prevents the results from being generalised to the entire adolescent population of Palestine. However, this limitation is perfectly managed. The authors justify it due to the difficult conditions for research in the region, openly declare it, and explain its consequences.

We appreciate the reviewer’s balanced evaluation of this limitation. We have made every effort to transparently report the use of convenience sampling, justify it within the challenging research context, and clearly state its implications for generalisability in the manuscript.

The ‘Discussion’ section is particularly robust. The authors not only report their findings, but also contextualise them within the global scientific literature, discussing both the evidence that supports them and that which contradicts them.

We are grateful for this encouraging feedback. Considerable effort was devoted to ensuring that the discussion provided a critical and balanced synthesis of the findings within the broader international literature.

The study is a very good example of rigorous and ethical research in an environment with logistical constraints.

We sincerely thank the reviewer for this thoughtful and affirming comment. Conducting rigorous and ethical research under challenging conditions was a central priority of this study, and we greatly appreciate the reviewer’s recognition of this effort.

Reviewer #2: Thank you for the opportunity to review this manuscript. It represents a commendable effort, and the article is generally well written and clearly communicated. I found it an engaging read. I do, however, have several important observations that I believe the authors should address to further strengthen the work.

We thank the reviewer for positive and encouraging comments on the manuscript.

Most of the concerns regarding this article stem from the limited information provided to evaluate the extent of bias introduced, particularly given the impossibility of randomization in the sample selection. This last consideration is a common issue in some attempts to establish population metrics, and it is well justified in this case in the text.

We thank the reviewer for this thoughtful observation. We acknowledge that convenience sampling introduces potential selection bias. To mitigate this, participants were recruited from diverse settings across the West Bank (community centers and social media platforms) to enhance demographic and geographic representation within the constraints of political instability and restricted institutional access. As the reviewer notes, such limitations are common in conflict-affected settings where probability-based sampling is not feasible, as justified in the Methods section. These limitations are clearly outlined in the manuscript, and we emphasized that the findings can’t be generalized to the broader Palestinian population.

Although the final sample size is more than double the initially estimated target, the risk of substantial bias remains considerable. The authors acknowledge several potential sources of bias in their limitations section; however, they do not provide sufficient data to assess the magnitude of these issues, among others.

We thank the reviewer for this comment. we agree with the reviewer that a large sample size does not eliminate the risk of bias. Formal estimation of bias was not possible because the study relied on non-probability sampling and no population registry or sampling frame was available for comparison with non-participants. This limitation is now clarified in the Limitations section, and findings are interpreted cautiously.

• Participation bias. The sampling strategy appears to favor individuals with auditory problems, which could significantly distort the representativeness of the findings. This potential bias is not discussed when comparing the results with other studies that may have relied on more representative sampling strategies.

Response: We agree that participation bias is a potential limitation. Participation bias is discussed in the limitations section of the revised manuscript.

• Access-related participation bias. A greater likelihood of participation among individuals with easier access to digital platforms introduces another layer of bias, potentially confounding, in an epidemiological fashion, the reported associations and limiting the external validity of the findings.

Response: We agree that individuals' accessibility to digital platforms might be variable, and this bias is also added to the limitations section in the revised manuscript.

• Lack of quality control. No procedures are described to verify the identity or characteristics of respondents. While this is a recognized challenge in open surveys, it should be explicitly addressed, particularly given that the study population is narrowly defined in terms of age.

Response: We agree that the lack of identity confirmation may impact the participants' recruitment quality control, even though it may facilitate participants' approval to participate. We have included this in the limitations section. Identity confirmation was not applicable in our study setting; the precision of age-specific findings requires future studies with stricter verification protocols.

• Reflection about the instrument. Although I was not previously familiar with this instrument, it appears to have been widely applied across different settings. However, several methodological aspects require clarification. Was the instrument reverse-translated to ensure linguistic accuracy? Was a pilot study conducted prior to full implementation? In addition, given the length of the questionnaire, how did the authors account for the potential bias arising from respondent fatigue, whereby answers toward the end may be less accurate or consistent?

Response: To minimize respondent fatigue, the questionnaire was structured to allow participants to pause and resume, reducing the likelihood of inconsistent responses toward the end.

The questionnaire was an Arabic translation version of ESIT-SQ, which was used as a study tool in a recent Palestinian study, the questionnaire underwent a forward-backward translation process to ensure language accuracy in prior research.

A pilot study was executed before the comprehensive data collection to evaluate the clarity, relevance, and feasibility of the questionnaire. The pilot research data were excluded from the final analysis.

• Survey outreach. An estimation of the total population reached through different recruitment strategies would be valuable for evaluating coverage and representativeness.

• Attrition and completion rates. The manuscript does not report the number of participants who completed the survey relative to the number of attempts. It is implausible that all individuals who began the survey completed it. Similarly, it is unclear whether all data fields were fully completed, or whether the survey platform required mandatory responses to proceed. Clarifying this aspect would be essential, as attrition rates and missing data patterns are key indicators of data quality and potential bias.

Response: To prevent attrition and incomplete responses, all fields of the manuscript were mandatory, and only complete responses could be submitted. Thus, the response rate calculation was not applicable.

In summary, while the sample size achieved is commendable, the absence of detailed information on recruitment, response validation, and attrition undermines confidence in the robustness and representativeness of the findings.

The presentation of results is excessively detailed, which diminishes the reader’s ability to discern the specific contributions of the article. Given that the topic is described as multifactorial and that the supporting evidence for associations with diverse risk factors remains generally weak, the simultaneous testing of a large number of variables is problematic. Moreover, some analyses based on the absence of associations within the dataset are presented but not adequately developed in the discussion. I recommend that the authors streamline their results by emphasizing those factors that appear most compelling or most closely aligned with the evidence introduced in the background, thereby strengthening the coherence and contribution of the manuscript.

We appreciate the reviewer's suggestion. Given the exploratory nature of the study and the topic's varied background, we attempted to maintain a full presentation of data to ensure transparency and prevent selective reporting. Both significant and non-significant findings were considered instructive and relevant to current research. The Discussion section concentrates on the factors that are most closely related to earlier evidence, allowing for easier interpretation while keeping the overall results.

Related to the previous point and regarding to the limited robustness of the sample, the use of modeling strategies may be problematic. The article would benefit from adopting a more explicitly exploratory tone, consistently avoiding the implication that the findings are representative of the entire population. For example, the results should be systematically framed as applying in “this sample of young Palestinians”, or something similar, rather than presented as generalizable estimates. In this light, the modeling exercises can still be considered valuable, but their contribution should be interpreted as illustrative and exploratory, rather than as evidence of definitive or population-level veracity.

We completely agree that, given the sample's constraints, the study should take a more clearly exploratory approach. The phrasing in the revised manuscript has been carefully altered to minimize implications of population-level generalizability. Results are now regularly presented as applicable to "this sample of young Palestinians" rather than the general population.

There appears to be a systematic error in how prevalence percentages are calculated and communicated. For example, the second paragraph of the Discussion states that girls have a higher prevalence than boys, yet the percentages reported (32.7% and 14.3%) are inconsistent with the tabulated data. Using the authors’ own figures—730 girls in total (Table 1), of whom 370 have tinnitus (Table 6)—the prevalence among girls should be 370/730 ≈ 50.7%, not 32.7%. This suggests the denominator used was the total sample rather than the sex-specific subgroup. Please carefully review all other variables to ensure this error is not repeated, and re-evaluate whether comparisons with external studies remain valid in light of the corrected estimates.

Response: Thank you for your notice. The mentioned numbers were based on calculations using the total sample as a denominator. However, it is more logical to use the sex specific subgroup number. Numbers have been revised, and proper corrections have been made.

Reviewer #3: The partial consideration of tinnitus as a psychosocial symptom is striking, given the context, both in the introduction and the discussion. The allusion to the healthcare costs of the problem is taken out of context and is not revisited in the discussion; it might be better to remove it.

We thank the reviewer for this insightful comment. We agree that tinnitus should be conceptualized within a broader psychosocial framework. Accordingly, we have revised both the introduction and the discussion by adding a paragraph to each section that more explicitly acknowledges the psychosocial dimensions of tinnitus. (Page 3/ Introduction, Page 39/Discussion).

In addition, as healthcare costs were not analyzed in the present study and were not revisited in the discussion, we have removed the paragraph referring to the economic burden of tinnitus from the introduction to improve coherence and maintain focus on the study objectives
