# Supplementary material for: Prevalence and associated risk factors of tinnitus among Palestinian adolescents aged 15–18: A cross-sectional study
Source: PLoS One. 2026 Mar 9;21(3):e0344420. doi: 10.1371/journal.pone.0344420 (PMC12970918; doi:10.1371/journal.pone.0344420)
Supplement: Attachment — Submitted filename: 14-02-2026 Responses.docx [file pone.0344420.s005.docx]

Reviewer #2: I’d like to thank the authors for their thoughtful revision of the text. This revised version shows clear effort to improve the manuscript, and several points raised in the initial review have been addressed in a satisfactory manner.

I appreciate the clearer acknowledgment of the study’s exploratory nature and tone, the more explicit discussion of limitations related to the sampling strategy, and the expanded description of the questionnaire (i.e. prior validation, pilot testing)

Some other comments appear to have been addressed mainly at a declarative level. While additional limitations are now mentioned, their implications are not always incorporated into the interpretation of results or comparisons with other studies. This does not detract substantially from the manuscript.

However, I would like to draw attention to one specific issue that remains unresolved and requires further careful revision. Although the authors state that prevalence calculations were corrected, the revised manuscript continues to report sex-specific prevalence percentages using the total sample as the denominator rather than sex-specific denominators. For instance, the proportion of females with tinnitus is still reported as 32.7% (370/1131) in both the abstract and Table 6, whereas the sex-specific prevalence would be approximately 50.7% (370/730), as correctly reflected in the discussion section.

While reporting percentages using the total sample as the denominator in a “2×2 table” format can be acceptable if clearly justified and consistently communicated, the use of these percentages in the abstract is potentially misleading. Moreover, when comparing the tables in the revised manuscript with those in the original version, I do not observe any changes to the numerical values. This suggests that, beyond revisiting the calculations in the tables themselves, it is important to carefully review all related interpretations and summaries of the data to ensure consistency and accuracy throughout the manuscript.

**Response: The percentages were revised and corrected using the sex-specific denominators rather than the total sample.**

While this appears to be a technical oversight, correcting it is important, as it affects subgroup comparisons and the interpretation of the findings. I therefore encourage the authors to revisit all prevalence calculations, ensure that appropriate denominators are used throughout, and update any related interpretations accordingly.

Overall, the manuscript has improved. and addressing this remaining issue would further strengthen its internal consistency and clarity.

Reviewer #3: Two comments regarding the insertions made in the introduction and conclusions, in response to my observations on the first version of the manuscript.

(1) Highlighting the greater (but marginal) risk attributed to the age of 17 years is delicate. From a developmental perspective, adolescent development encompasses different classifications; one of the most traditional distinguishes adolescence into early, middle, and late age ranges. The article with which this study is compared and which purportedly supports its findings (ref. 20) compares age ranges (11–14 years and 14–18 years) rather than single-year ages. Moreover, the single-year ages of 15 and 17 years, which are used as points of comparison in the present study, are included within the same age range of middle adolescence from a developmental standpoint, both evolutionarily and in the cited study. This is relevant because the use of age ranges is developmentally grounded in the understanding that adolescents are undergoing continuous development, without absolute thresholds, within a phase of the life cycle that has been relatively understudied and whose upper age limit has been extended (Baird, S., Choonara, S., Azzopardi, P. S., Banati, P., Bessant, J., Biermann, O., Capon, A., Claeson, M., Collins, P. Y., De Wet-Billings, N., Dogra, S., Dong, Y., Francis, K. L., Gebrekristos, L. T., Groves, A. K., Hay, S. I., Imbago-Jácome, D., Jenkins, A. P., Kabiru, C. W., … Viner, R. M. (2025)). In addition, from a technical standpoint, the statistical test applied to the age variable in the present study evaluates the statistical hypothesis of difference versus no difference across the set of ages as a whole, rather than differences between specific single-year ages.

**Thank you for providing this important and constructive remark. We completely agree that interpreting age-related data in adolescent necessitates careful conceptual and methodological considerations. As a result, we have tempered the terminology in discussion sections to avoid the impression that age 17 represents a discrete developmental category. Instead, we refer to the data as a marginal variance observed throughout middle adolescence, rather than evidence of a discrete age-specific risk. We have also included a brief line in the Discussion to acknowledge modern perspectives that emphasize adolescence's flexibility and stretched upper border (e.g., Baird et al., 2025), emphasizing the absence of hard developmental thresholds.**

(2) Reference 58 acknowledges contextual elements, including collective violence (I suggest review : World Health Organization. (2004). Preventing violence: A guide to implementing the recommendations of the World report on violence and health (ISBN: 9241592079). World Health Organization. Page 1) as a risk factor for PTSD, in which tinnitus would be an associated symptom of a broader clinical condition. In light of this background, I would like to invite the authors to think critically on the investigative, ethical, and epistemic perspective that challenges us all to question the value, energy, and level of detail involved in studying tinnitu as a specific symptom in relation to the adverse childhood experiences (ACEs or Adverse Childhood Events) that adolescents—understood as members of the species in a critical developmental phase that shapes their personal and societal futures—are experiencing in contexts that would make it desirable to consider variables not captured by standardized instruments developed in other settings, as frequently occurs in countries with levels of development different from those considered “developed.”

**Response: Thank you for this comment. We agree with the importance of situating tinnitus within the broader clinical and contextual framework of adolescent health, as tinnitus may represent an associated symptom of conditions such as PTSD, particularly in contexts marked by collective violence and adverse childhood experiences (ACEs). In response, we have included this point in the discussion and the suggested reference has been included.**

**The sentence included in the discussion: Moreover, while tinnitus emerged as a reported symptom, it should be understood within the broader context of trauma-related conditions such as PTSD, particularly in conflict-affected areas such as Palestine, where the population, including all age groups, is frequently experiencing collective violence and adverse childhood experiences (ACEs)**
